# Feed intake, preferences, in vivo digestibility, and nutritional value of tedera (*B. bituminosa var*. *bituminosa*) and maralfalfa (*Pennisetum purpureum*) in Canary sheep

**DOI:** 10.1007/s11250-025-04284-z

**Published:** 2025-01-31

**Authors:** M. Barbera, V. Mendoza-Grimón, J. Espinosa, E. Rodriguez-Ponce, A. Gracia, E. SanJuán, M. R. Ventura

**Affiliations:** 1 Department of Animal Science, University of Las Palmas de Gran Canaria, Arucas, Spain; 2 Instituto de Investigación IUNAT, Grupo GEOVOL, Universidad de Las Palmas de Gran Canaria, Las Palmas de Gran Canaria, Spain

**Keywords:** Canary sheep, Feed intake, Digestibility, Preference, Tedera, Maralfalfa

## Abstract

This study evaluated the nutritional value and energy content of tedera (*B. bituminosa var. bituminosa*) and maralfalfa (*Pennisetum purpureum*) through analyses of chemical composition, digestibility, intake, and preference trials. Tedera was compared with maralfalfa and alfalfa hay (*Medicago sativa*). Tedera showed higher crude protein (193 g CP/kg DM) and estimated energy (10.5 MJ DE/kg DM) but lower dry matter (286.3 g DM/kg) and neutral detergent fiber (373 g NDF/kg DM) than both maralfalfa and alfalfa hay. The in vitro organic matter digestibility (IVOMD) of tedera was 61.7%, compared to 51.0% for alfalfa hay and 66.3% for maralfalfa. Digestible organic matter (DOM) ranged from 467 g/kg DM in alfalfa hay to 566.4 g/kg DM in tedera. Four Canary sheep with a mean body weight (BW) of 42.2 ± 5.0 kg were used for digestibility and preference trials. The live weights of the sheep were recorded at the start and end of the 12-day trial. Feed offered and refusals were weighed and recorded daily for eight days, while feces were collected for four days to calculate apparent in vivo digestibility. For tedera, the apparent in vivo OM digestibility, estimated digestible energy, and digestible organic matter were 69.4%, 11.8 MJ/kg DM, and 637.7 g/kg DM, respectively. Preference and feed intake were compared between tedera, maralfalfa and alfalfa hay. Total DM consumption was 1091.3 g/day (tedera + maralfalfa + alfalfa hay), with alfalfa hay intake representing 40.8%, maralfalfa 37.3%, and tedera 21.9% of the total DM consumed. However, no significant differences were observed in the ratio of forages consumed/offered (44.8% for tedera and 51.8% for maralfalfa) or in the total grams of DM, CP, and MJ/kg of DE consumed by the sheep with both forages. The sheep adopted different feeding strategies in response to the chemical composition and nutritive value of the forages. Preferences and intake in this trial were associated with high NDF content in maralfalfa and alfalfa hay and with the high CP content in tedera rather than digestibility results. This may be due to the complementarity of the three forages and the higher CP content in tedera affecting intake. Nevertheless, tedera and marafalfa could be a good forage considering its nutritive value, digestibility, and proven growth performance in herbivores.

## Introduction

Tedera (*Bituminaria bituminosa C.H. Stirton var. bituminosa*) is a perennial legume valued as an essential forage in semi-arid regions due to its adaptability and productivity in dry climates and poor soils, as well as its ability to retain green leaves year-round (Real et al. 2018; Oldham et al. 2015). Tedera has agronomic characteristics suitable for Mediterranean-like climates, providing high-quality green forage for grazing animals or as cut-and-carry feed during summer and autumn (Real et al. 2018). Its productive and nutritional potential has led to its use in feeding ruminants in various regions, including the Canary Islands (hand feeding), Australia (grazing), and Mediterranean countries (Real et al. 2014; Foster et al. 2015; Ventura et al. 2009). Studies have shown that sheep maintain good health when fed fresh tedera exclusively, sustaining their live weight and body condition score (Oldham et al. 2015; Real et al. 2018). Additionally, tedera has been evaluated for intake, growth performance, and use in various forms, including grazing, fresh feed, and hay, in poultry, goats, and sheep (Real et al. 2018; Ventura et al. 2009; Barberá et al. 2019; Oldham et al. 2015).

Historically, tedera has been considered low in palatability due to secondary compounds such as tannins, alkaloids, coumarins, saponins, and oxalates, some of which emit strong odors that reduce its palatability and intake (Pecetti et al. 2007, 2015; Ventura et al. 2019; Ghaffari et al. 2015; Tava et al. 2007). However, the nutritional value of tedera, in terms of chemical composition and digestibility, has been proven to be similar or even superior to other legumes (Oldham et al. 2015; Pecetti et al. 2007; Ventura et al. 2019). Conversely, maralfalfa (*Pennisetum purpureum*) is a perennial fodder grass introduced to the Canary Islands for animal feed due to its dry matter productivity and nutritive value (Palacios et al. 2013).

In addition to productivity and nutritive value, intake, palatability, and forage preferences significantly impact the efficient production of animal products in herbivores. When animals consume palatable feed with good nutritional value, their growth performance and production (e.g., milk and meat) improve. While familiarity and habits play crucial roles in feed preference (Wallis et al. 2014; Thomas et al. 2010), animals may not consume large quantities of forage even after becoming accustomed to its taste (Real et al. 2018). This behavior can be influenced by factors such as nutritive value, chemical composition, or nutritional deficiencies. Also, certain substances, such as alkaloids, tannins, furanocoumarins, phenolic derivatives, and sulfur compounds in legumes, can act as antinutritional factors and reducing preferences, palatibility and intake (Real et al. 2018; Ventura et al. 2000, 2009, 2019; Pecetti et al. 2015; Ghaffari et al. 2015; Tava et al. 2022).

Several studies support the hypothesis that sheep's preference for annual legumes is related to their chemical characteristics, with sheep tending to select plants with higher nutritional value (Thomas et al. 2010; Ventura et al. 2000; Pecetti et al. 2007; Tava et al. 2022; Mayland and Shewmaker 1999). Consequently, herbivores often avoid specific vegetation components associated with plant maturity or low nutritional value due to amino acid imbalances or fiber content. Herbivores dynamically adjust their diet in response to the quality, both physical and chemical composition, of available forage (Provenza et al. 1996).

While clear predictive relationships for voluntary forage intake from diverse grasslands remain elusive, voluntary intake may be influenced by factors such as seasonality and nutritional value, as observed in studies on goats feeding on tedera (Real et al. 2018; Ventura et al. 2009). Voluntary intake often correlates with dry-matter digestibility, structural carbohydrate content, preferences, and overall palatability (Thomas et al. 2010; Bruinenberg et al. 2002; Blaxter et al. 1966). However, the digestibility, crude protein, energy concentration, and voluntary intake of a feed collectively determine its global nutritional value. Greenhalgh and Reid (1971) suggest that the primary factor controlling roughage intake in ruminants is the maximum rate at which the food can be digested. Additionally, Blaxter et al. (1966) and Blaxter and Wainman (1961) propose that the rate of roughage digestion is often positively correlated with its digestibility, implying that intake may be influenced by this factor.

Considering this background, the main objective of this study was to compare the chemical composition, intake, preferences, and digestibility of two tropical forages, tedera and maralfalfa, as well as alfalfa hay. Although previous studies have investigated the nutritional value of these forages, this study aims to provide a comparative analysis of tedera and maralfalfa concerning intake, preferences, and digestibility. Alfalfa hay was included as a reference forage widely used in the Canary Islands. The hypothesis was that these forages exhibit different characteristics, nutritional values, and digestibility, leading to varying responses in terms of preference and intake by animals. Identifying these differences will help predict which forage offers the best nutritional value and intake.

## Material and methods

### Location, animals, and feeding management

The study was conducted at the experimental farm of the Veterinary College of Las Palmas de Gran Canaria University (Canary Islands, Spain) (latitude 27° 55′ 45″; longitude 15° 23′ 20″), where Canary breed sheep were housed. The climate is subtropical, with average annual temperatures ranging from 18 °C to 24 °C (64.4°F to 75.2°F) and precipitation between 150 and 600 mm.

Four sheep were used in this study. They were housed in individual pens (190 × 165 cm) equipped with feeders and water devices. One week before the adaptation period, all animals were dewormed with Ivermectin (Ivomec® Injectable, Boehringer Ingelheim, España), administered subcutaneously at a dose of 200 µg per kg of body weight (1 ml per 50 kg).

This study complies with the guidelines of the European Union Council (2010/63/EU) for the use of experimental animals.

The forage species used in this study were tedera (*Bituminaria bituminosa var. bituminosa*), maralfalfa (*Pennisetum purpureum*), and alfalfa hay (*Medicago sativa*). The tedera was randomly selected from spontaneous populations that had developed without irrigation or fertilization and was collected in April at the maturity stage of flowering. The maralfalfa was harvested from the Granja Agrícola Experimental (Cabildo de Gran Canaria) in Arucas, Canary Islands (latitude 27° 55′ 45″; longitude 15° 23′ 20″), where it was grown under irrigation and fertilization. The alfalfa hay used was commercial hay. Approximately 6 kg/day of each variety (tedera, maralfalfa, and alfalfa hay) were collected over eight days, and samples from different plants of each type were pooled for analysis to study the digestibility, preference, and intake of these forages.

### Analytical methods

Forage samples were weighed, cut, and dried at 60 °C to approximately 90% dry matter (DM). They were then ground to pass a 1-mm screen for chemical analyses and a 2-mm screen for in vitro digestibility (IVD) tests. DM, ash, and crude protein (CP) were determined according to standard methods described by the Association of Official Analytical Chemists (AOAC 2000) (methods 930.15, 942.05, and 976.05, respectively). Additionally, ash-free neutral detergent fiber (NDFom) was determined using sodium sulfite in the neutral detergent (ND) following the method of Goeríng and Van Soest (1970) with an ANKOM 220 Fiber Analyzer (Ankom Technology Corporation).

In vitro dry matter digestibility (IVDMD) and organic matter digestibility (IVOMD) were determined using the two-stage pepsin-cellulose method (Pepcel) (Aufrere 1982). Digestible energy (DE) was estimated as 0.0185 × IVOMD (NRC 1988).

### Preference and intake trials

The preference and feed intake of fresh tedera were determined using four male sheep (Canary breed) with a mean body weight (BW) of 42 ± 5.0 kg and aged 11 months. Maralfalfa and alfalfa hay were used to compare feed intake and preference. Before the study, the sheep were adapted to the forages by feeding them tedera, maralfalfa, and alfalfa hay for seven days. After this adaptation period, the sheep were offered experimental diets for eight consecutive days in the morning (10:00 a.m.) to test their preferences for the different forages: tedera, maralfalfa, and alfalfa hay. During the first three days (Trial 1), the intake and preferences for all three forages were studied. For the last five days (Trial 2), the preference was evaluated only for tedera and maralfalfa.

The sheep were housed individually in pens for eight days, each with free access to feeders. Each feeder offered 1 kg of fresh forage daily. In Trial 1, each pen contained three feeders: one with 1 kg of fresh tedera, one with 1 kg of fresh maralfalfa, and one with 1 kg of alfalfa hay. In Trial 2, the preferences between tedera and maralfalfa were compared. Therefore, the sheep had daily access to two feeders: one with 1 kg of fresh tedera and the other with 1 kg of fresh maralfalfa.

In each trial, daily refusals and intakes were weighed and recorded for each feeder. The daily feed intake was calculated by the difference between the amounts offered and refused. Proportional consumption was determined as the ratio (g/g) of the average daily DM intake consumed to the DM offered for tedera, alfalfa hay, and maralfalfa. Preference for one forage was defined as the percentage of total intake derived from that forage (Meier et al. 2012).

Daily average CP, NDF, and DE intake per animal were estimated from the total eight-day measurement period and determined as follows: DE (MJ/animal/day) = Digestible energy feed (MJ/kg DM) × DM intake (kg/animal/day). Similarly, daily average CP intake (CPI, g/animal/day) was calculated as CP (g/day) × DM intake (kg/animal/day), and NDF intake (g/animal/day) was calculated as NDF (g/day) × DM intake (kg/animal/day) (García-Trujillo and Caceres 1984).

### In vivo digestibility and nutritive value of tedera

The study on the apparent in vivo digestibility of tedera was conducted with four sheep over a four-day trial period using the traditional in vivo method (Khan et al.^ 2003^), with the sheep housed in metabolism cages. Canary-breed sheep were selected as experimental animals. Four males with a similar initial body weight (BW, 42.50 ± 5.0 kg, 11 months of age) were individually weighed and housed in individual metabolic cages (height × length × width, 160 × 120 × 60 mm). Each cage was equipped with water and feeders, along with clean trays and tubs for the separate collection of feces.

Before the study, the sheep were adapted to the forage by feeding them tedera for seven days. After the adaptation period, the sheep were offered tedera for four consecutive days in the morning (10:00 a.m.), with each day's offering being 15% more than the daily intake of forage from the previous day. Refusals of tedera and feces were weighed each morning before a new ration was distributed. Samples (100 g) were identified and stored at − 20 °C. Composite samples of the collected material were dried at 60 °C to a constant weight in a ventilated oven, ground to pass a 1-mm screen, and stored in sealed plastic containers for subsequent analyses. The contents of DM were analyzed for feed and feces, and feed intake was calculated. Accurate records of feed intake, refusals, and fecal output were maintained. The apparent in vivo digestibility of tedera was determined from feed intakes and feces according to García-Trujillo and Caceres (1984): Apparent in vivo digestibility = 100 x (intake—excreted)/intake.

### Statistical analysis

The data were analyzed using analyses of variance (ANOVA) with the SPSS statistical package (version 27) employing the Generalized Linear Model. The model incorporated factors such as forage type, individual animals, days, and their interactions. To identify significant differences, Tukey’s method was applied, with a significance level set at *p* = 0.05. Additionally, multiple linear regression analyses were conducted to examine the relationships between intake preference and various factors including chemical composition (NDF, CP), digestibility, and DE of the forages.

## Results

### Chemical composition and nutritional value of the forages

The chemical composition of the forages used in this study is presented in Table 1. The dry matter (DM) content in tedera and maralfalfa ranged from 286.3 to 903 g/kg DM of fresh forage, with organic matter (OM) content between 793 and 918 g/kg DM. Maralfalfa had a lower OM content, while there were no significant differences between alfalfa hay and tedera. Crude protein (CP) content ranged from 116 g/kg DM to 193 g/kg DM, with tedera having the highest CP content. Neutral detergent fiber (NDF) and acid detergent fiber (ADF) contents ranged from 373 to 576 g/kg DM and 247 to 357 g/kg DM, respectively, with both being lower in tedera forage.

**Table 1:** Dry matter, chemical components, and nutritive value of Tedera, Maralfalfa, and Alfalfa

	Tedera	Maralfalfa	Alfalfa hay
Dry matter (g/kg)	286.3	316	903
Organic matter (g/kg DM)	918	793	916
Crude protein (g/kg DM)	193	146	116
Neutral detergent fiber (g/kg DM)	373	571	576
Acid detergent fiber (g/kg DM)	247	261	357
In vitro			
Organic matter digestibility (%)	61.7	66.3	51.0
Digestible organic matter (g/kg DM)	566.4	525	467
Estimated digestible energy (MJ/kg DM)	10.5	9.7	8.6

In vitro organic matter digestibility (IVOMD) ranged from 51.0% in alfalfa hay to 66.3% in maralfalfa. The in vitro digestible organic matter (DOM) ranged between 467 and 566.4 g/kg DM, with tedera showing higher DOM content and alfalfa hay showing the lowest. Additionally, digestible energy (DE) ranged from 8.6 to 10.5 MJ/kg DM, being highest in tedera and lowest in alfalfa hay.

### Preference and intake forages

#### Trial 1: Preference and intake between tedera, maralfalfa, and alfalfa hay

Table 2 presents the dry matter (DM), crude protein (CP), neutral detergent fiber (NDF, expressed in g/DM), and digestible energy (DE, expressed in MJ/DE) consumed by sheep fed tedera, maralfalfa, and alfalfa hay, together with the requirements according to the NRC (2007), and the percentage of the different nutrients covered with the intake of the different forages.

**Table 2:** Daily feed intake, requirements, % of covered and proportional consumption of forages (Tedera, Maralfalfa, and Alfalfa Hay): Dry Matter (DM), Intake, Consumed Crude Protein (CP), Consumed Neutral Detergent Fiber (NDF) expressed as g/DM, and Consumed Digestible Energy (DE) expressed as MJ/kg DM. Ratio consumed/offered (g/g): Ratio (g/g) of averages of daily DM intake consumed and DM offered

	Tedera		Marafalfa		Alfalfa hay		Total Intake	Requirements*	% Covered
	mean	std	mean	std	mean	std			
Offered (g DM)	818.7	216.45	899.2	147.09	969.6	247.04	2.688		
Intake (g/DM)	238.8^b^	67.83	407.1^ab^	71.32	445.4^a^	191.53	1.091	1.180	93
Consumed CP (g/DM)	46.1	13.09	59.4	10.41	51.7	22.22	157.2	142	110
Consumed NDF(g/DM)	89.8^b^	25.5	232.4^a b^	40.72	256.6^a^	110.32	578.8	354	163
Consumed DE (MJ/DE)	2.7	0.77	3.9	0.69	3.8	1.65	10.4	14.4	72
Proportional consumption: Consumed/offered									
Ratio (%)	22.2^b^		38.0^a^		39.4^a^				

Different letters show significant differences at α 0.05

*According to NRC (2007) for male sheep of 42+/−5 kg of live weight and a daily gain expected of 100 g

Finally, the ratio between consumed and offered forage (expressed in %) is shown. The intake range of forages varied significantly, from 238.8 g DM for tedera to 445 g DM for alfalfa hay. There were notable differences in NDF intake, ranging from 89.8 g NDF for tedera to 256.6 g NDF for alfalfa hay.The CP intake by the sheep was 46.1 g for tedera, 59.4 g for maralfalfa, and 51.7 g for alfalfa hay. DE intake ranged from 2.7 MJ/kg DM for tedera to 3.9 MJ/kg DM for maralfalfa. Despite these differences, the CP and DE content did not show significant variations between the forages. The consumed/offered ratio ranged from 22.2% to 39.4%, with higher values for alfalfa hay and maralfalfa, showing no significant differences between them, but significant differences compared to tedera.

The total DM consumed was 1091.3 g/day, with tedera representing 21.9%, maralfalfa representing 37.3%, and alfalfa hay representing 40.8% of the total DM consumed. This corresponded to an NDF intake of 15.5% from tedera, 40.1% from maralfalfa, and 44.3% from alfalfa hay, out of the total NDF consumed (578 g). Additionally, the total CP and DE consumed daily were 157.2 g and 10.4 MJ/kg DM, respectively. There were no significant differences in the percentage of CP consumed from each forage, which ranged from 29.3% to 37.8%, and the percentage of DE consumed, which ranged from 25.9% to 37.5% of the total forages consumed daily. We can observe that according to the NRC (2007) requirements for sheep, with the total intake of forages, the consumed CP and NDF is greater than their requirements (110% of the CP and 163% NDF requirements covered), while the DM (93%) and energy (72%) were less than their requirements.

**Fig. 1:**
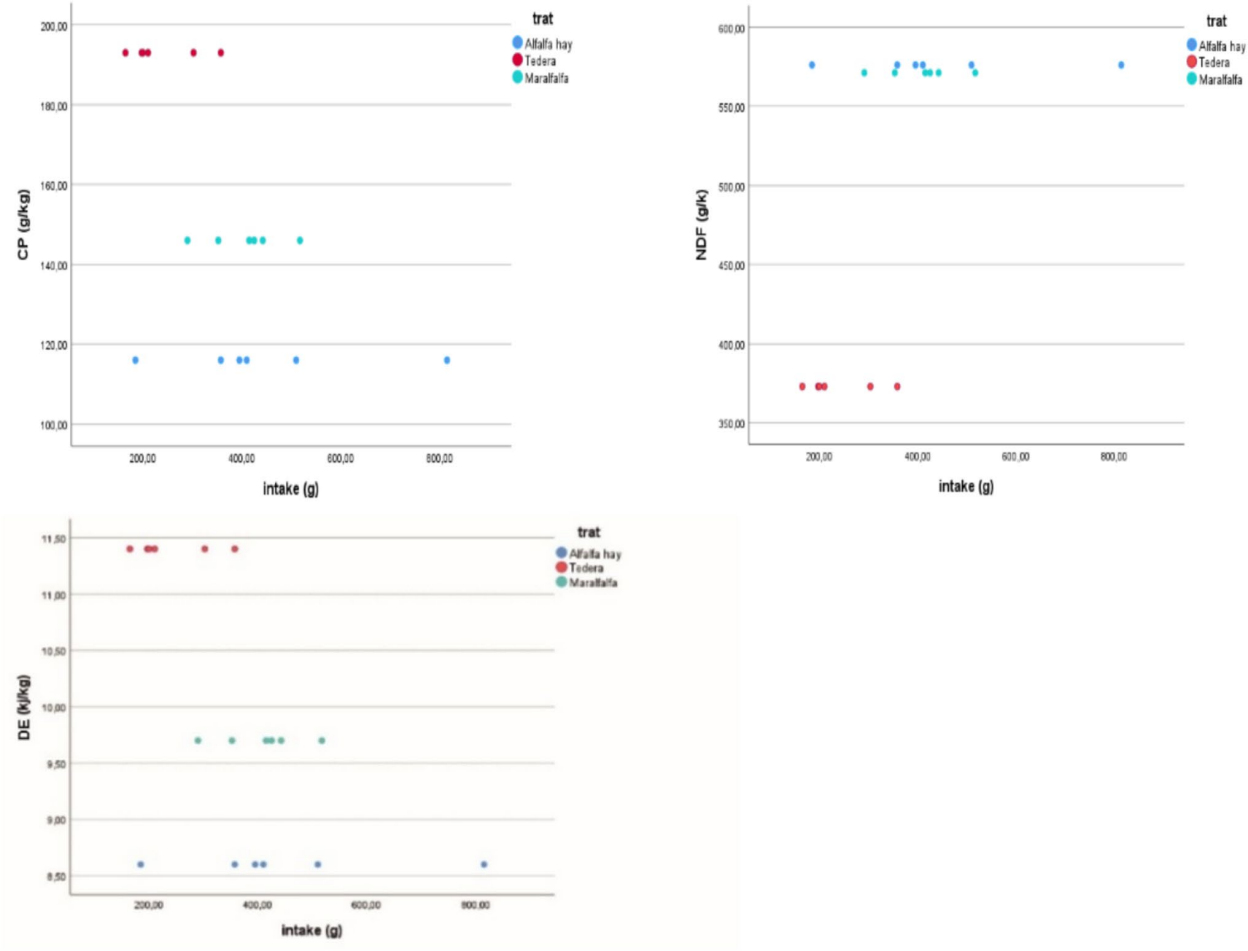
Relationship between intake (g) and Crude Protein (CP), Neutral Detergent Fiber (NDF) and Digestible Energy (DE) expressed as g/DM and MJ /kg DM respectively for different forages

**Fig. 2:**
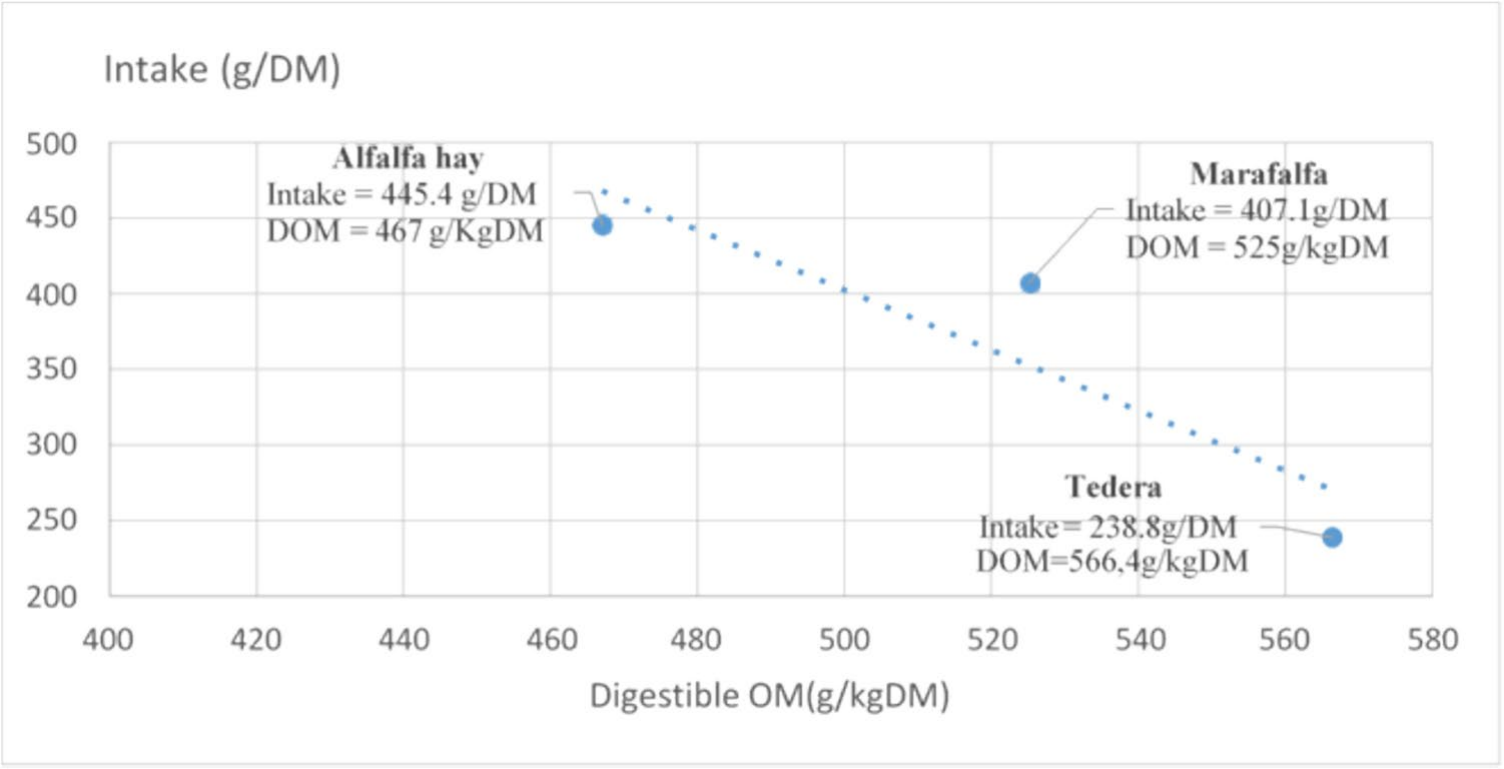
Relationship between intake (g) and digestible organic matter (DOM) expressed as g/DM for different forages

Figures 1 and 2 illustrate the relationship between forage intake and the content of CP, NDF, DE, and digestible organic matter (DOM). Tedera forage exhibited the highest contents of CP and DE (Fig. 1), but its NDF content and g/DM intake were lower than those of the other two forages. In contrast, maralfalfa and alfalfa hay had lower CP and DE contents but higher NDF contents compared to tedera. Despite tedera's superior CP, energy content, and digestibility, it resulted in a lower intake.

Regarding the relationship between intake (g) and digestible organic matter (DOM) shown in Fig. 2, DOM is highest in tedera and lowest in alfalfa hay. There is a tendency for intake to increase as the DOM of the forages decreases. Consequently, tedera has a lower intake despite its higher DOM, compared to alfalfa hay, which has the highest intake and the lowest DOM among the three forages.

#### Trial 2: Preference and intake between tedera and maralfalfa

Table 3 presents the comparison of intake, including dry matter (DM), crude protein (CP), neutral detergent fiber (NDF), and digestible energy (DE, MJ/kg DM) consumed by sheep fed tedera and maralfalfa, along with the ratio (%) of forages consumed/offered in Trial 2. The results indicate that the daily mean intake of tedera and maralfalfa was 444.0 g/DM and 688.2 g/DM, respectively, showing a significant difference (p < 0.05) between these forages. Differences were observed in the NDF intake, where sheep consumed 166.9 g NDF from tedera and 392.9 g NDF from maralfalfa. Furthermore, the CP intake by sheep was 85.7 g from tedera and 100.4 g from maralfalfa. The estimated DE consumed was 5 MJ/kg DM from tedera and 6.6 MJ/kg DM from maralfalfa but, CP and DE consumed did not show significant differences between forages. Despite this, no significant differences were observed in the ratio of forages consumed/offered, which was 44.8% for tedera and 51.8% for maralfalfa, indicating no significant differences between them.

**Table 3:** Daily feed intake and proportional consumption of forages (Tedera, Maralfalfa). Dry Matter (DM), Crude Protein (CP), Neutral Detergent Fiber (NDF) expressed as g/DM, and Digestible Energy (DE) expressed as MJ/kg DM. Ratio consumed/offered (g/g): Ratio (g/g) of averages of daily DM intake consumed and DM offered

	Tedera		Maralfalfa		Total Intake	Requirements*	%Covered
	mean	std	mean	std			
Offered (g DM)	998.2	73	1327.2	174.85			
Intake (g DM)	444.0^b^	154.85	688.2^a^	142.28	1132	1.180	96
Consumed CP (g DM)	85.7	29.89	100.4	20.77	186.1	142	131
Consumed NDF (g DM)	166.9^b^	58.22	392.9^a^	81.24	560	354	158
Consumed DE (M/kg DM)	5.0	1.77	6.6	1.38	11.6	14.4	81.2
Proportional consumption: Consumed/offered							
Ratio (%)	44.8		51.8				

Different letters show significant differences at α 0.05

*According to NRC (2007) for male sheep of 42+/−5 kg of live weight and a daily gain expected of 100 g

In Trial 2, the total dry matter (DM) consumed (tedera + maralfalfa) was 1.132 g/day. Tedera accounted for 39.2% of the total DM consumed, while maralfalfa represented 60.7%. This resulted in a total neutral detergent fiber (NDF) intake of 559.9 g, with 29.9% coming from tedera and 70.1% from maralfalfa. Furthermore, the total crude protein (CP) consumed daily was 186.1 g, and the total digestible energy (DE) consumed daily was 11.6 MJ/kg DM. There were no differences in the percentage of CP consumed from each forage, which ranged from 46% in tedera to 54% in maralfalfa. Similarly, the percentage of DE consumed ranged from 42.5% in tedera to 57.5% in maralfalfa of the total forages consumed daily.

According to the NRC (2007) requirements for sheep, with the total intake of tedera and marafalfa, the CP and NDF consumed was greater than their requirements (131% of the CP and 158% of the NDF requirements), while the percentages of DM (96%) and Energy (81.2%) were less than the requirements.

### Digestibility in vitro of tedera and maralfalfa

The in vitro digestibility results for tedera and maralfalfa are presented in Table 4. The in vitro digestibility of tedera and maralfalfa was 61.7% and 66.3% respectively. The digestible organic matter (OM) was 566.4 g/kg DM for tedera and 525.4 g/kg DM for maralfalfa.

**Table 4:** Comparison of digestibility in vitro expressed as (%), Digestible Organic Matter (OM), Digestible Crude Protein (CP), Digestible Neutral Detergent Fiber (NDF) expressed as g/kg DM, and estimated digestible energy, expressed as MJ/kg DM of Tedera and Maralfalfa

		Tedera		Maralfalfa	
		mean	std	mean	std
Digestibility in vitro (%)		61.7	2.4	66.3	2.1
Digestible OM (g/kg DM)		566.4^a^	21.8	525. 4^b^	16.9
Estimated DE (MJ/kg DM)		10.5	0.4	9.7	0.3
Digestible CP (g/kg DM)		119^a^	4.2	96.8^b^	2.5
Digestible NDF (g/kg DM)		230.1^b^	8.1	378.7^a^	9.6

Different letters show significant differences at α 0.05

Considering these results, the estimated digestible energy (DE) was 10.5 MJ/kg DM for tedera and 9.7 MJ/kg DM for maralfalfa. The digestible crude protein (CP) was 119 g/kg DM for tedera and 96.8 g/kg DM for maralfalfa, and the digestible neutral detergent fiber (NDF) was 230.1 g/kg DM for tedera and 378.7 g/kg DM for maralfalfa. Although there were no significant differences in in vitro digestibility and estimated DE between the two forages, significant differences were observed in the digestible OM, CP, and NDF values.

### Digestibility in vivo of tedera

The results of the apparent in vivo digestibility of tedera are presented in Table 5. The in vivo digestibility was found to be 69.4%, and a digestible organic matter (OM) content of 637.7 g/kg DM. Based on these results, the estimated digestible energy (DE) was 11.8 MJ/kg DM, the digestible (CP) in tedera was 133 g/kg DM, and the digestible (NDF) was 258.8 g/kg DM.

**Table 5:** Digestibility (%), estimated digestible energy expressed as MJ/kg DM, Digestible Organic Matter (OM), Digestible Crude Protein (CP), and Digestible Neutral Detergent Fiber (NDF) expressed as (g/kg DM), in vivo* and *in vitro of Tedera

		mean	std
Digestibility (%)	in vivo	69.4^a^	9.54
	in vitro	61.7^b^	2.38
Estimated DE (MJ/kg DM)	in vivo	11.8	1.6
	in vitro	10.5	0.4
Digestible OM (g/kg DM)	in vivo	637.7^a^	87.56
	in vitro	566.4^b^	21.85
Digestible CP (g/kg DM)	in vivo	133	13
Digestible NDF (g/kg DM)	in vivo	258.8	16.8

Different letters show significant differences at α 0.05

#### Comparison of in vivo and in vitro digestibility of tedera

Table 5 compares the in vitro and in vivo digestibility of tedera fodder. There are significant differences (*p* < 0.05) in the percentage of digestibility and the digestible OM (g/kg DM) between the in vivo and in vitro methods for tedera. However, the estimated DE (MJ/kg DM) does not show significant differences between the two methods of determining digestibility.

## Discussion

The primary objective of this study was to compare the nutritional value of tedera, maralfalfa, and alfalfa hay, and secondary, to determine their relationship with preference and intake. Thomas et al. (2010) mentioned that sheep prefer certain legumes or forages based on their chemical characteristics, selecting plants with higher nutritive value. Our analysis of the nutritional value revealed that, although the DM content in tedera was lower than in the other forages, tedera had higher OM, CP, DOM, and DE compared to maralfalfa and alfalfa hay. However, the NDF content in tedera was lower than in the other forages.

Comparing our results with those of other authors, the chemical composition of tedera aligns with the ranges reviewed by Kaymak et al. (2021) and Ventura et al. (2004), who reported tedera hay content ranging from 10.3% to 20.4% for CP, 23.8% to 41.9% for ADF, and between 38% and 56.3% for NDF. Additionally, the digestibility rate fluctuated between 46 and 62% for different tedera varieties (Pecetti et al. 2007; Sternberg et al. 2006). Based on these results, one might expect a better intake for tedera. According to studies by Bruinenberg et al. (2002) and Thomas et al. (2010), sheep should prefer this forage over the others, as preferences and intake are related to their chemical characteristics. However, in Trial 1, comparing the preferences for tedera, maralfalfa, and alfalfa hay, the sheep consumed more maralfalfa (37.3%) and alfalfa hay (40.8%) than tedera (21.9%). Similarly, in Trial 2, comparing the preference between tedera and maralfalfa, tedera represented 39.2% of the total fodder consumed, while maralfalfa represented 60.7%, contributing 70.1% of the total NDF consumed. In both trials, the animals consumed forages that covered CP and NDF requirements in excess but did not cover the total requirements of ED and DM according to the NRC (2007).

The most significant difference between these forages (maralfalfa and alfalfa hay) and tedera was the high content of NDF and ADF in the former and the higher protein content in Tedera. Our study found a relationship between the DM amount ingested by the sheep and the NDF content in the forages. Sheep consumed more forages with higher NDF content, such as alfalfa hay and maralfalfa. However, there were no differences in the percentages provided from each forage for CP and DE consumed daily (Table 2). Similarly, Thomas et al. (2010) and Mendes et al. (2010) found that NDF was positively correlated with preference, while ADF was negatively correlated at the vegetative stage.

However, sheep preferred forages with lower NDF, higher digestibility, and higher nitrogen at the senesced stage of the plants. Perhaps because as plants aged, ADF became a vital determinant of preference, suggesting that animals avoid plants with high proportions of indigestible components as plants age.

Other research has found that diet selection in sheep may be influenced by the rates of digestion of carbohydrates and proteins in their diet. Sheep select between two forages based on crude protein content, avoiding excess crude protein. The proportion of crude protein selected depends on the carbohydrate sources in the feeds, being lower when the carbohydrate source in both feeds was rapidly rather than slowly fermented (Kyriazakis and Oldham 1997; Cooper et al. 1995). As observed in the present work, animals avoid an excess of CP (< tedera consumption) in their diet, if they are given the opportunity to choose between several forages. Therefore, the animals do not eat different plants randomly. Their feeding is highly selective, consuming forage to meet their nutritional requirements by selecting various combinations of plants (Thomas et al. 2010; Blaxter et al. 1966).

The energy concentration of food and its digestibility also determine nutritional value and can be related to preferences and intake. Bruinenberg et al. (2002) and Thomas et al. (2010) stated that while voluntary intake is often related to structural carbohydrate content, preferences and the intake of roughage may be related to digestibility.

In our study, we observed that the in vitro digestibility was lower in alfalfa hay and higher in maralfalfa compared to tedera, but without differences justifying the preference for maralfalfa and alfalfa hay over tedera (Table 1). While DOM is higher in tedera and lower in alfalfa hay, intake tends to be higher when the DOM of the forages is lower (Fig. 2). Thus, tedera intake is lower despite its higher DOM compared to the high intake of alfalfa hay, which has the lowest DOM of the three forages. This suggests that with better digestibility, the animal needs to eat less forage, as reported by Thomas et al. (2010), who noted that sheep prefer plants with higher digestibility, particularly at the senesced stage. Additionally, the higher digestible NDF of maralfalfa supports the hypothesis of Mendes et al. 2010 that fiber content plays a necessary role in the ingestion and preference of sheep. Even so, it seems more realistic to consider the complementarity of the three forages rather than focusing on a single indicator (digestibility, CP, NDF, or ADF considered separately), as the total intake of DM and total nutrients obtained is the sum of the three forages.

Despite that, the in vitro digestibility of tedera falls within the OM digestibility range (mean = 65.2%) described by Hardy et al. (2023), analyzing seven accessions of tedera, but it is lower than the results found by Fernández-Habas et al. (2022), with a mean digestibility of 86.2% for three varieties of *Bituminaria bituminosa* under water treatment, determined by near-infrared spectroscopy (NIRS). Further research is needed to compare in vitro methods of analyzing digestibility in legumes, such as NIRS and enzymatic methods.

On the other hand, the apparent in vivo OM digestibility of tedera with Canary sheep averaged 69.4%, the digestible organic matter was 637.7 g/kg DM, and the estimated digestible energy was 11.8 MJ/kg DM. In vivo OM digestibility was higher than the results reported by Oldham et al. (2015) for Merino sheep fed fresh tedera (59%) and alfalfa hay (55%). These differences could be due to the animals, the stage of maturity of the plants (Thomas et al. 2010), or the varieties of tedera fed by Oldham et al. (2015) (*albomarginata* and *crassiuscula* varieties). Finally, comparing the apparent in vivo digestibility of tedera with its in vitro digestibility (Table 5), the in vivo result was significantly higher, suggesting that in vitro digestibility underestimates the real digestibility of the fodder. Further research is needed to compare in vitro methods of analyzing digestibility.

In conclusion, while tedera has higher crude protein, energy content, and digestibility, it resulted in lower preference. This counterintuitive finding may be due to (a) the complementarity of the forages, with sheep reaching their limit of CP intake and thus preferring forages with lower CP content; (b) the role of fiber content in the ingestion and preference of the sheep for specific forages or (c) the presence of secondary compounds in tedera that limit intake. Perhaps in other studies it would be necessary to increase the number of days of the trials, the number of animals used and make analyses of secondary compounds that could better clarify the preferences and consumption of these forages.

## Conclusions

Sheep adopt different feeding strategies in response to changes in plant characteristics, chemical composition, and the nutritive value of forages. While preference and intake of forages were related to NDF and CP content, it is crucial to consider the complementarity of the three forages rather than focusing on a single trait such as digestibility, CP, NDF, or ADF. Additionally, the presence of certain secondary compounds in tedera may limit its intake. Despite this, tedera and marafalfa shows potential as a good forage due to its high nutritive value, including crude protein, energy content, and digestibility, along with proven good growth performance when used to feed herbivores. Further studies are needed to confirm these findings and fully understand the factors influencing forage preference and intake in sheep.

## Data Availability

Data that support the findings of this study are available from the corresponding author, MRV, upon request.
